# Buccinator myomucosal flap for the treatment of velopharyngeal insufficiency in patients with cleft palate and/or lip^☆^^☆☆^

**DOI:** 10.1016/j.bjorl.2017.08.006

**Published:** 2017-09-12

**Authors:** Rafael Denadai, Anelise Sabbag, Cassio Eduardo Raposo Amaral, João Carlos Pereira Filho, Mirian Hideko Nagae, Cesar Augusto Raposo Amaral

**Affiliations:** a Hospital SOBRAPAR, Instituto de Cirurgia Plástica Craniofacial, Campinas, SP, Brazil; b Universidade Estadual de Campinas (UNICAMP), Faculdade de Ciências Médicas, Departamento de Desenvolvimento Humano e Reabilitação, Campinas, SP, Brazil

**Keywords:** Cleft palate, Velopharyngeal insufficiency, Hypernasality, Buccinator myomucosal flap, Fissura palatina, Insuficiência velofaríngea, Hipernasalidade, Retalho miomucoso do músculo bucinador

## Abstract

**Introduction:**

The interpretation of the speech results obtained with the buccinator myomucosal flap in the treatment of velopharyngeal insufficiency in patients with cleft palate has been limited by the restriction in the number of patients and the time of postoperative follow-up.

**Objective:**

To evaluate the effect of the buccinator myomucosal flap on speech hypernasality in the treatment of patients with cleft palate and velopharyngeal insufficiency.

**Methods:**

Patients with repaired cleft palate (± lip) who were submitted to surgical correction of velopharyngeal insufficiency using the bilateral buccinator myomucosal flap were assessed. Hypernasality (scores 0 [absent], 1 [mild], 2 [moderate], or 3 [severe]) was analyzed by three evaluators by measuring the audiovisual records collected in early and late preoperative and postoperative periods (3 and 12 months, respectively). The values were considered significant for a 95% Confidence Interval (*p* < 0.05).

**Results:**

Thirty-seven patients with cleft palate (± lip) showing moderate (16.2%) or severe (83.8%) hypernasality in the preoperative period were included. Analyses of the late postoperative period showed that hypernasality (0.5 ± 0.7) was significantly (*p* < 0.05) lower than the hypernasality of the preoperative and recent postoperative periods (2.8 ± 0.4 and 1.7 ± 0.9, respectively).

**Conclusion:**

The buccinator myomucosal flap is effective in reducing/eliminating hypernasality in patients with cleft palate (± lip) and velopharyngeal insufficiency.

## Resumo

**Introdução:**

A interpretação dos resultados de fala obtidos com o retalho miomucoso do músculo bucinador no tratamento da insuficiência velofaríngea em pacientes fissurados tem sido limitada pela restrição do número de pacientes e do tempo de seguimento pós-operatório.

**Objetivo:**

Avaliar o efeito do retalho miomucoso do músculo bucinador sobre a hipernasalidade da fala no tratamento de pacientes fissurados com insuficiência velofaríngea.

**Método:**

Foram avaliados pacientes com fissura palatina (± lábio) reparada, com retalho miomucoso do músculo bucinador bilateral para a correção cirúrgica da insuficiência velofaríngea. A hipernasalidade (escores 0 [ausente], 1 [leve], 2 [moderada] ou 3 [severa]) foi analisada por três avaliadores por meio da mensuração dos registros audiovisuais coletados nos períodos pré-operatório e pós-operatórios (3 e 12 meses, respectivamente). Os valores foram considerados significativos para um intervalo de confiança de 95% (*p* < 0,05).

**Resultado:**

Foram incluídos 37 pacientes fissurados com hipernasalidade moderada (16,2%) ou severa (83,8%) no período pré-operatório. As análises do período pós-operatório tardio revelaram que a hipernasalidade (0,5 ± 0,7) foi significativamente (*p* < 0,05) menor do que a hipernasalidade dos períodos pré-operatório e pós-operatório recente (2,8 ± 0,4 e 1,7 ± 0,9; respectivamente).

**Conclusão:**

O retalho miomucoso do músculo bucinador é eficaz na redução/eliminação da hipernasalidade nos pacientes fissurados com insuficiência velofaríngea.

## Introduction

Approximately 5–36% of patients with cleft lip and palate submitted to primary palatoplasty have post-operative velopharyngeal insufficiency, a structural defect characterized by the inability to attain complete closure of the velopharyngeal sphincter (i.e., there is a remaining space between the posterior pharyngeal wall and the soft palate after its maximum excursion during speech, routinely referred to as “gap”) due to mechanical restriction, inappropriate positioning and/or tissue insufficiency.^1^, ^2^ Velopharyngeal insufficiency results in mandatory and compensatory speech disorders that impair the overall quality of life and the interpersonal relationships of patients with clef palate.^1^, ^2^ Among the mandatory speech disorders, hypernasality is considered the most representative symptom and is defined as an excessive nasal resonance during oral sound production.^1^, ^2^

The surgical treatment of velopharyngeal insufficiency is a challenge for well-trained surgeons, due to the broad spectrum of patients’ clinical presentation and lack of consensus on the optimal surgical approach.^3^ The choice between the most often used surgical procedures (sphincteroplasty, pharyngeal flaps, double reverse z-plasty or intravelar veloplasty) has been based mainly on the size and pattern of the velopharyngeal gap.^3^, ^4^, ^5^, ^6^, ^7^, ^8^, ^9^, ^10^

Although satisfactory speech results have been described with such surgical interventions,^3^, ^4^, ^5^, ^6^, ^7^, ^8^, ^9^, ^10^ some factors (e.g., types of previously performed palatine surgeries, presence of scarring on the palate, and medium or large velopharyngeal gap) limit the applicability of double reverse z-plasty or intravelar veloplasty to a small number of patients.^3^, ^6^, ^7^, ^8^, ^9^, ^10^

Moreover, sphincteroplasty and pharyngeal flaps are associated with relevant complications, such as obstructive sleep apnea, snoring, oral breathing, hyponasal speech, and death^11^; however, it is relevant to emphasize that it was recently demonstrated that the presence of pharyngeal flap in middle-aged individuals was not a risk factor for obstructive sleep apnea.^12^

In this context, palatoplasty for the elongation of the palatine veil using the myomucosal flap of the buccinator muscle has been performed with the aim of normalizing the velopharyngeal function and minimizing the obstructive airway sequelae.^13^, ^14^, ^15^, ^16^, ^17^, ^18^ However, the small number of patients, the heterogeneity of the assessed groups, and the limited postoperative follow-up have restricted the conclusions related to speech and hypernasality results of this specific surgical approach.^13^, ^14^, ^15^, ^16^, ^17^, ^18^

Particularly in Brazil, Bozola et al.^19^ were the first to describe the detailed anatomy of the buccal flap, calling it buccinator myomucosal flap. Franco et al.^20^ reported on the versatility of this flap for palatal reconstruction of patients without cleft lip and palate. Additionally, Raposo do Amaral^15^ showed a preliminary experiment on the use of this flap for the treatment of velopharyngeal insufficiency, particularly in patients with cleft lip and/or palate. However, only the data related to the three-month postoperative evolution of the velopharyngeal gap were detailed.

Therefore, the objective of this study was to evaluate the effect of bilateral buccinator myomucosal flap on speech hypernasality in the treatment of patients with cleft palate (±lip) and velopharyngeal insufficiency.

## Methods

This is a prospective study of all patients with cleft palate ± lip consecutively submitted to bilateral buccinator myomucosal flap for the treatment of velopharyngeal insufficiency between January 2010 and January 2014 by a single surgeon. Demographic, clinical (type of cleft and characteristics of the speech hypernasality and velopharyngeal gap^1^, ^2^, ^21^, ^22^, ^23^), and surgical data (prior unsuccessful surgical treatment of velopharyngeal insufficiency, width of the buccinator myomucosal flap and complications) and speech hypernasality results^7^, ^8^, ^24^, ^25^, ^26^, ^27^ were collected through standardized assessment tools by the same multiprofessional team. Patients with hearing loss (>25 decibels), craniofacial syndromes, submucosal cleft palate, cleft palate without surgical correction and/or incomplete postoperative follow-up (<12 months) were excluded from the study.

The study was approved by the Hospital Ethics Committee (001/15) and is in accordance with the 1975 Helsinki Declaration, reviewed in 1983. All patients included in the study accepted to participate by signing the free and informed consent form.

### Velopharyngeal function assessment

All patients were included in the multidisciplinary institutional protocol of perceptual-auditory and instrumental evaluations (nasopharyngoscopy examinations) of velopharyngeal function. Standardized audiovisual recordings (speech recordings and nasopharyngoscopy examinations) were systematically performed in the preoperative and postoperative periods at 3 and 12 months (early and late, respectively). All parameters were tested using a defined number of phonetically-balanced words and phrases.

After a diagnosis of velopharyngeal insufficiency was attained based on previously established perceptual-auditory speech assessment,^1^, ^2^, ^21^ the patients were investigated through nasopharyngoscopy examinations performed by a plastic surgeon, together with the team of speech-language pathologists. During the nasopharyngoscopy, the palate nasal surface was inspected to define the sagittal or transverse orientation of the palatine veil elevator muscle.^22^, ^23^

The size of the velopharyngeal gap during maximum closure in speech was characterized as: complete velopharyngeal closure; punctiform (demonstrated by bubbling); small (velopharyngeal closure >80% and <100%); medium (velopharyngeal closure between 50% and 80%); or large (velopharyngeal closure <50%).^9^, ^10^, ^22^ Velopharyngeal gap patterns were defined as coronal, sagittal or circular, with or without the presence of the Passavant fold.^23^

### Surgical approach

**Figure 1:**
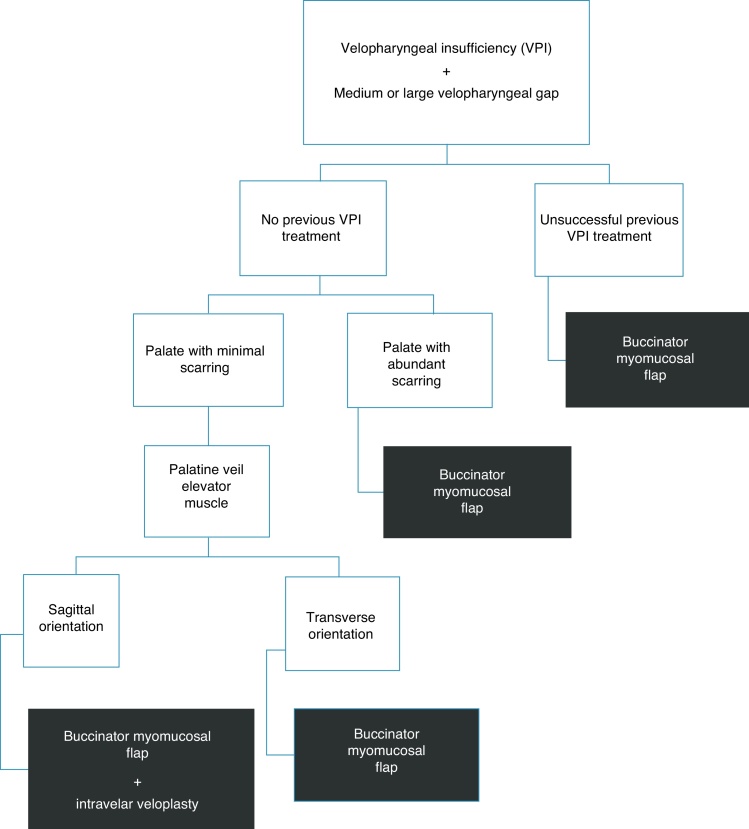
Therapeutic algorithm for the treatment of velopharyngeal insufficiency of patients submitted to cleft palate correction surgery, without palatine fistula and with medium or large velopharyngeal gap.

In our center, a therapeutic algorithm has been used for the surgical treatment of patients with cleft palate ± lip with velopharyngeal insufficiency since 2010. Our therapeutic rationale was based on the following criteria: prior unsuccessful surgical treatment (palatoplasty with pushback technique, radical dissection of the palatine veil musculature or double reverse z-plasty) of velopharyngeal insufficiency; scarring in the transition region between the hard and soft palates evaluated by oroscopy; and orientation of the palatine veil elevator muscle and velopharyngeal gap size evaluated by nasopharyngoscopy (Fig. 1).

In summary, patients with prior unsuccessful surgical treatment of velopharyngeal insufficiency were submitted to the buccinator flap (secondary or tertiary surgeries). Patients with no prior surgical treatment of velopharyngeal insufficiency with large amount of scarring on the palate or with minimal scarring on the palate and transverse orientation of the palatine veil elevator muscle were submitted to the buccinator flap surgery (primary surgeries). Patients with no previous velopharyngeal insufficiency, with minimal scars on the palate and sagittal orientation of the palatine veil elevator muscle were submitted to the buccinator flap surgery associated with intravelar veloplasty (primary surgeries). The velopharyngeal gap pattern was not a determinant in our surgical approach. Only the subgroup of patients submitted to the buccinator myomucosal flap was included in this study.

### Surgical technique

**Figure 2:**
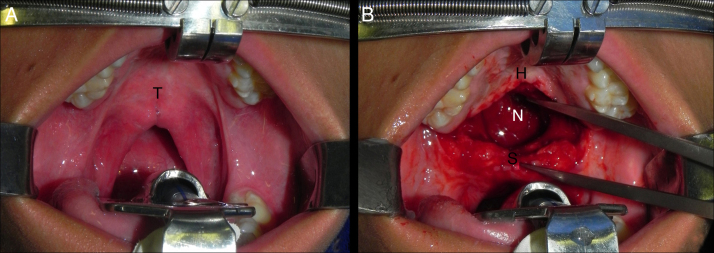
A, Intraoperative intra-oral photograph showing the scars at the junction of the hard and soft palate. B, Intraoperative intra-oral photograph demonstrating the defect created between the hard and soft palates after radical detachment of the palatine veil and mobilization of the soft palate toward the posterior pharyngeal wall (“T”, transition between hard and soft palates; H, hard palate; “S”, soft palate; “N”, nasopharynx).

The bilateral buccinator myomucosal flap was used for palatine elongation as recommended by Maeda et al.^28^ Surgeries were performed with the patient in horizontal dorsal decubitus, with orotracheal intubation and under general anesthesia. After local infiltration (0.9% saline solution and adrenaline 1: 100,000), the transition between the hard and soft palates was incised and dissected, allowing mobilization of the soft palate toward the posterior pharyngeal wall and creating a defect between the hard and soft palates (Fig. 2). Other surgical maneuvers (e.g., dissection of the major palatine vascular-nervous bundle and/or palatine veil musculature including the pterygoid hamulus) to allow the retropositioning of the soft palate without tension were performed depending on soft tissue availability and/or magnitude of local cicatricial retraction.

**Figure 3:**
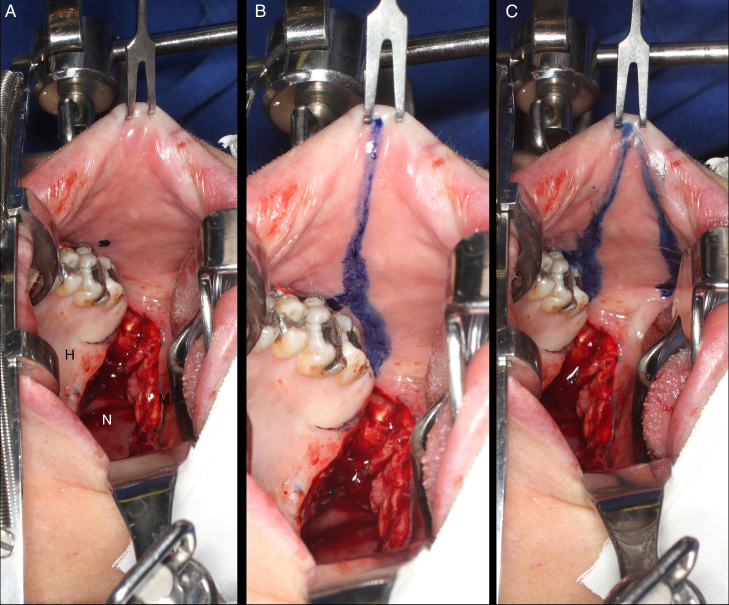
Intraoral photographs of the demarcation and creation of the buccinator myomucosal flap. A, Marking of the Stensen duct exit with methylene blue. B, Delimitation of the cranial portion passing just below the Stensen duct exit. C, Delimitation of the caudal portion parallel to the cranial portion and the anterior portion near the oral opening (“H”, hard palate; “S”, soft palate; “N”, nasopharynx).

**Figure 4:**
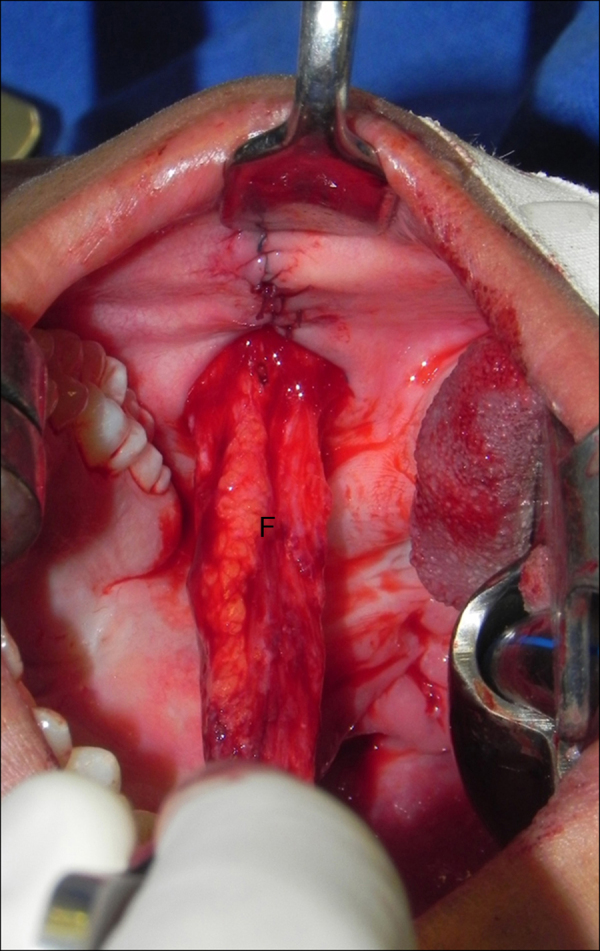
Intraoral photograph showing the buccinator myomucosal flap and synthesis of the donor area without tension (“F”, buccinator flap).

**Figure 5:**
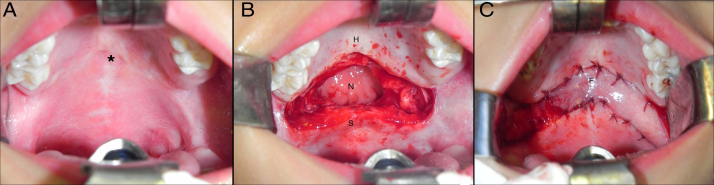
Intraoperative photographs showing: A, palatal scars; B, the defect created between the hard and soft palate and C, the reconstruction of the defect with the bilateral buccinator myomucosal flap (*, scars; “H”, hard palate; “S”, soft palate; “N”, nasopharynx; “F”, buccinator flap).

Intravelar veloplasty was performed in patients with sagittal orientation of the palatine veil elevator muscle the with the aim of anatomically repositioning the palatine musculature and, consequently, contributing to the palatine veil mobilization. The buccinator flaps were then delimited with methylene blue in the medial (jugal mucosa), cranial (passing just below the Stensen duct exit), caudal (parallel to the cranial), anterior (ending in “V” close to the oral opening) and posterior (pedicle established near the third molars) portions (Fig. 3). The distance between the cranial and caudal lines (width of the flap) was defined by the measurement of the defect size created in the palatal transition. The myomucosal flaps containing the buccinator muscles were carefully dissected so as not to disrupt the buccal fat pad. The flap on the left was flipped to reconstruct the nasal mucosa, whereas the flap on the right was rotated and advanced to reconstruct the oral mucosa. The mucosa of the donor sites was directly sutured (polyglactin 910 4-0 suture) (Figure 4, Figure 5). Three to six weeks after surgery, the pedicles were divided if there was difficulty chewing and/or limitation in mouth opening.

#### Measurement of surgical results

All early and late preoperative and postoperative records were analyzed by three evaluators experienced in the treatment of patients with cleft lip and palate in a random, independent and blinded manner (without prior knowledge of the assessed patients and/or periods). Speech results were evaluated based on a structurally correctable variable and distributed according to a widely adopted scoring scale^8^, ^24^, ^25^, ^26^, ^27^: hypernasality (0 = none; 1 = mild; 2 = moderate; or 3 = severe). The reduction of at least one level of severity classification in the early and late postoperative periods was considered an improvement in hypernasality.^7^

Obstructive sleep apnea screening tests (STOP-Bang questionnaire [range 0–8; scores 0–2, 3–4 and ≥5 defined as low risk, intermediate risk and high risk, respectively] and Epworth sleepiness scale [range 0–24; scores <11 and ≥11 defined as low risk and high risk]^29^, ^30^, ^31^, ^32^ were thoroughly applied by the multidisciplinary team in the early and late preoperative and postoperative periods.

### Statistical analysis

For the descriptive analysis, the mean was used for metric variables and the percentages for categorical variables. The tests ANOVA, *χ*^2^, Yates’ Correction, Equality of Two Proportions and Confidence Interval for the Mean were used for all comparative analyses. The degree of agreement between the evaluators was analyzed using kappa values and was considered excellent (kappa values ≥ 0.89) for the analyzed variables. The sample size estimate was 33 patients (*α* = 0.05; *β* = 0.12). The Statistical Package for Social Sciences, version 20 (SPSS, Chicago, IL, USA) and Minitab version 16 (Minitab, Inc., USA) were used for statistical analysis. The values were considered significant for a Confidence Interval of 95% (*p* < 0.05).

## Results

Thirty-seven patients (mean age 20.8 years) were included. The sample consisted mainly (*p* < 0.05) of patients with unilateral incisor transforaminal cleft, submitted to prior unsuccessful surgical treatment of velopharyngeal insufficiency (Table 1). A total of 37 bilateral buccinator myomucosal flaps were used for the treatment of patients with large and circular velopharyngeal gaps and severe hypernasality (Table 2, Table 3). All patients had transient edema in the donor areas. There were 11 (29.7%) complications related to surgical procedures (3 [27.3%] cases of dehiscence that healed spontaneously, 3 [27.3%] mouth opening limitations resolved with pedicle division, 2 [18.2%] cases of hematoma in donor areas that were surgically drained; 2 [18.2%] cases of partial necrosis of the distal portions of the flaps used for reconstruction of the oral mucosa that healed spontaneously without residual sequelae; and 1 [9%] fistula at the transition between the hard and soft palates treated with tongue flap after data collection was completed) (Table 1). All screening tests for obstructive sleep apnea were classified (*p* > 0.05) as low risk in the recent and late preoperative and postoperative periods (Table 2).

**Table 1:** Characteristics of patients with cleft palate and velopharyngeal insufficiency treated with buccinator myomucosal flap (*n* = 37).

Characteristics	Patients (*n* = 37)	*p*-Value
*Age (years) M* *±* *SD*	20.8 ± 12.4 (5–41)	–
*Female/male n (%)*	16 (43.2)/21 (56.8)	>0.05

*Spina classification n (%)*		
Incomplete post-foramen incisor cleft	2 (5.4)	<0.01
Complete post-foramen incisor cleft	10 (27)	
Unilateral incisor transforaminal cleft	14 (37.8)	
Bilateral incisor transforaminal cleft	11 (29.7)	

*Origin of patients, n (%)*		
Treated initially in our service	20 (54.1)	>0.05
Treated initially in other services	17 (45.9)	

*Primary palatoplasty*^a^*n (%)*		
Recent (≤18 months)/late (>18 months)	19 (51.4)/18 (48.6)	>0.05

*Previous surgical treatment, n (%)*		
Palatine fistula (yes/no)	26 (70.3)/11 (29.7)	<0.01
Velopharyngeal insufficiency (yes^b^/no)	23 (62.2)/14 (37.8)	<0.03
Flap width (millimeters) *M* ± SD	15.5 ± 4.2	–

S*urgical complications, n (%)*		
Yes/no	11 (29.7)/26 (70.3)	<0.01

*n*, number of patients; *M*, mean; SD, standard deviation; VFI, velopharyngeal insufficiency; –, not applicable.

a Two-stage palatoplasty using the Goteborg technique (*n* = 14, 37.8%) and single-stage palatoplasty using Von Langenbeck, Veau, and Ward–Kilner techniques, with or without intravelar veloplasty (*n* = 23, 62.2%).

b All patients had failed prior surgical treatment (pushback palatoplasty, radical dissection of the palatine veil musculature, or double reverse z-plasty).

**Table 2:** Screening tests for obstructive sleep apnea in the preoperative and postoperative periods of velopharyngeal insufficiency treatment with the bilateral buccinator myomucosal flap (*n* = 37).

Characteristics	Preoperative	Postoperative		
		Recent (3 months)	Late (12 months)	*p*-Value^a^
STOP-Bang questionnaire *M* ± SD	0.73 ± 0.45	0.78 ± 0.42	0.73 ± 0.45	>0.05
Low risk, *n* (%)	37 (100)	37 (100)	37 (100)	>0.05
Intermediate risk, *n* (%)	0 (0)	0 (0)	0 (0)	
High risk, *n* (%)	0 (0)	0 (0)	0 (0)	
Epworth sleepiness scale *M* ± SD	3.59 ± 1.79	3.62 ± 1.66	3.57 ± 1.68	>0.05
Low risk, *n* (%)	37 (100)	37 (100)	37 (100)	>0.05
High risk, *n* (%)	0 (0)	0 (0)	0 (0)	

*n*, number of patients; *M*, mean; SD, standard deviation.

a Inter-period comparisons.

**Table 3:** Velopharyngeal gap size and pattern in the preoperative and postoperative periods (*n* = 37).

Characteristics of the velopharyngeal gap	Preoperative	*p*-Value^a^	Postoperative				*p*-Value^b^
			Recent (3 months)	*p*-Value^a^	Late (12 months)	*p*-Value^a^	
*Gap pattern n (%)*							<0.01
Absent	0 (0)	<0.03	5 (13.5)	<0.01	21 (56.8)	<0.01	
Coronal	11 (29.7)		13 (35.1)		6 (16.2)		
Circular	15 (40.5)		13 (35.1)		7 (18.9)		
Circular with Passavant fold	11 (29.7)		6 (16.2)		3 (8.1)		
Sagittal	0 (0)		0 (0)		0 (0)		

*Gap size n (%)*							<0.01
Complete velopharyngeal closure	0 (0)	<0.02	5 (13.5)	<0.01	21 (56.8)	<0.01	
Punctiform	0 (0)		2 (5.4)		9 (24.3)		
Small	0 (0)		13 (35.1)		7 (18.9)		
Medium	10 (27)		12 (32.4)		0 (0)		
Large	27 (73)		5 (13.5)		0 (0)		

*n*, number of patients; –, not applicable.

a Intra-period comparisons.

b Inter-period comparisons.

### Velopharyngeal gap size and pattern

The comparative analyses between the postoperative periods showed a progressive reduction (early > late: *p* < 0.05) in the velopharyngeal gap size and a progressive increase (early < late: *p* < 0.05) in the number of patients with velopharyngeal gap pattern classified as absent. The velopharyngeal gap size measured in the early and late postoperative periods was significantly (*p* < 0.05) smaller than the preoperative evaluation (Table 3).

### Hypernasality

**Figure 6:**
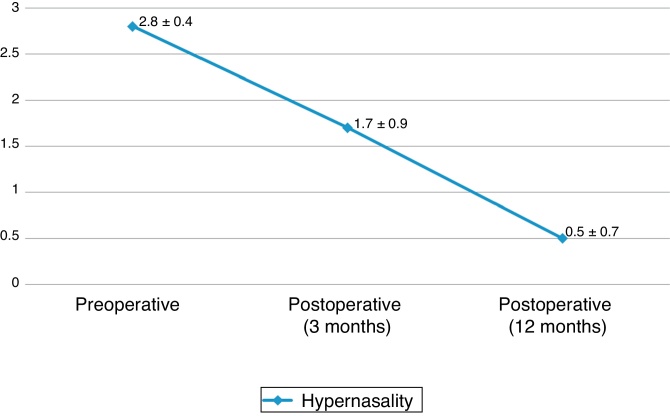
Distribution (mean ± standard deviation) of the hypernasality score (score 0–3) in the early and late preoperative and postoperative periods (3 and 12 months, respectively). Values of *p* < 0.01 for all comparisons (preoperative >3-month postoperative >12-month postoperative).

The comparative analyses between the postoperative periods showed a progressive reduction (early > late: *p* < 0.05) of hypernasality and a progressive increase (early < late: *p* < 0.05) in the number of patients with hypernasality improvement. The hypernasality measured in the early and late postoperative periods was significantly (*p* < 0.05) lower than the preoperative one (Table 4, Fig. 6). There was no hyponasality in this series.

**Table 4:** Characteristics of speech hypernasality in the preoperative and postoperative periods of velopharyngeal insufficiency treatment with bilateral buccinator myomucosal flap (*n* = 37).

Characteristics	Preoperative	*p*-Value^a^	Postoperative				*p*-Value^b^
			Recent (3 months)	*p*-Value^a^	Late (12 months)	*p*-Value^a^	
*Hypernasality n (%)*							
Improvement (yes/no)	–	–	28 (75.7)/9 (24.3)	<0.01	37 (100)/0 (0)	<0.01	<0.01
None	0 (0)	<0.01	4 (10.8)	<0.01	22 (59.5)	<0.01	<0.01
Mild	0 (0)		10 (27)		11 (29.7)		
Moderate	6 (16.2)		17 (45.9)		4 (10.8)		
Severe	31 (83.8)		6 (16.2)		0 (0)		

*n*, number of patients; –, not applicable.

a Intra-period comparisons.

b Inter-period comparisons. *Note*: The degree of agreement between the evaluators was considered excellent (kappa values ≥ 0.89) in all measurements.

## Discussion

In the present study, we evaluated a subgroup of patients with cleft palate (± lip) and with velopharyngeal insufficiency that were surgically treated with bilateral buccinator myomucosal flap and demonstrated a significant reduction in speech hypernasality in the early and late postoperative periods. After 12 months of follow-up, our rates of patients with hypernasality improvement (100%), of patients without hypernasality (59.5%) and patients with mild hypernasality (29.7%) was in agreement with those found in the specific literature on the buccinator myomucosal flap.^13^, ^14^, ^16^, ^17^, ^18^

However, any interpretations on the comparisons between our results and those reported in previous investigations should be carried out with caution, since there is a wide heterogeneity in sample sizes and composition, in the study designs, in the flap creation and in the methodologies applied when measuring the results.^13^, ^14^, ^16^, ^17^, ^18^

Since the first descriptions of the use of buccinator myomucosal flaps in patients with cleft palate,^19^ this flap has been applied mainly to primary palatoplasty or palatine fistula reconstruction.^28^, ^33^ In 2004, Hill et al.^18^ were the first to use this flap to lengthen the palate of patients with cleft palate and velopharyngeal insufficiency. Since then, particularly in the context of velopharyngeal insufficiency, studies^12^, ^13^, ^15^, ^16^ on the use of this flap have included patients with cleft palate and velopharyngeal insufficiency, in addition to concomitant palatine fistula.

Moreover, syndromic patients, patients with submucosal cleft palate and/or patients without cleft lip and palate (i.e., velopharyngeal insufficiency secondary to neoplasia or trauma sequelae) were also included.^16^ Only two studies^15^, ^18^ (*n* = 16 in both) included patients with cleft palate and isolated velopharyngeal insufficiency (i.e., without associated palatine fistula). However, only one group^18^ retrospectively analyzed speech results, while the other^15^ restricted the results in the classification of velopharyngeal gap size and pattern in the 3-month postoperative period.

In our study, patients with craniofacial syndrome, submucosal cleft palate and/or untreated palatine fistulas were excluded. Although this restricted the final sample included in the study, we reduced some biases, because the excluded subgroups have intrinsic aspects that confound the speech result measurements^1^, ^2^, ^3^, ^8^, ^9^, ^10^, ^25^ and, therefore, have been evaluated separately in different studies.^3^, ^8^, ^9^, ^10^, ^25^ Additionally, the present study is the first to analyze the results related to speech hypernasality after the use of this flap in the treatment of velopharyngeal insufficiency in the national literature.

The mean age of the included patients is older than the recommended age for the surgical treatment of velopharyngeal insufficiency, since interventions in patients between 4 and 12 years of age (variable according to the investigation) seem to be related to better speech results.^9^, ^10^, ^26^, ^34^, ^35^ Previous studies^13^, ^14^, ^15^, ^16^, ^17^, ^18^ with the buccinator myomucosal flap consisted mainly of pediatric patients with velopharyngeal insufficiency. Therefore, our data are complementary to those presented previously^13^, ^14^, ^15^, ^16^, ^17^, ^18^ since this flap was effective in the surgical treatment of older patients (including adults). The older age in our sample reflects the reality of the surgical treatment of velopharyngeal insufficiency in our center and others,^34^, ^35^, ^36^ since older patients have been referred (frequently adopted or from rural regions with low human development index) with unrepaired cleft palate or residual velopharyngeal insufficiency.

Several characteristics of the buccinator myomucosal flap (e.g., abundant buccinator muscle vascularization, pedicle location, arc of rotation, flap flexibility, tissue elasticity and minimal morbidity in the donor area) have been reported as advantages for its use in palate surgeries.^13^, ^14^, ^15^, ^16^, ^17^, ^18^, ^19^, ^20^ However, in addition to the intrinsic characteristics to each type of flap, aspects related to the velopharyngeal gap have also been considered in the choice of surgical approach, specifically for the treatment of patients with cleft palate and velopharyngeal insufficiency. In this context, most patients with cleft palate and a medium or large velopharyngeal gap have been treated with sphincteroplasty or pharyngeal flaps.^3^, ^4^, ^5^, ^7^, ^11^, ^24^, ^25^, ^37^

In 2005, Armor et al.^37^ demonstrated that sphincteroplasty and pharyngeal flaps provide the best speech results in coronal and noncoronal (circular or sagittal) velopharyngeal gap patterns, respectively. In our study and others,^13^, ^14^, ^15^, ^16^, ^17^, ^18^ the buccinator myomucosal flap was used regardless of the velopharyngeal gap pattern.

Thus, based on the velopharyngeal function improvement demonstrated here and in other studies,^13^, ^14^, ^15^, ^16^, ^17^, ^18^ the buccinator muscle flap has an additional advantage over the sphincteroplasty and the pharyngeal flaps, since it can be used in patients with medium or large gaps, regardless of the velopharyngeal gap pattern. There are previously described additional advantages,^13^, ^14^ which are reinforced by our findings: the surgical approach using the buccinator muscle flap is more anatomical and physiological, because when the palate is short, the anatomical defect is treated directly (i.e., the soft palate including the palatine veil musculature is dissected and retropositioned, with consequent elongation of the palatine veil) without affecting the posterior pharyngeal wall; absence of postoperative hyponasality; and absence of airway obstruction, since there is no myomucosal tissue bridge between the posterior pharyngeal wall and the soft palate, as occurs with pharyngeal flaps.

As our patients were classified as low risk for obstructive sleep apnea in the measurements performed in the preoperative and early and late postoperative periods, polysomnography examinations were not performed. This reasoning has also been adopted in other studies^13^, ^14^, ^16^, ^38^ on velopharyngeal insufficiency and has been established in some questionnaires and clinical models for obstructive sleep apnea screening.^29^, ^30^, ^31^, ^32^ We performed the obstructive sleep apnea screening by applying tests (STOP-Bang questionnaire and Epworth sleepiness scale) that have been previously validated in Brazil.^29^, ^30^ Although these questionnaires have been widely applied as tools for obstructive sleep apnea screening with high specificity and/or sensitivity,^29^, ^30^, ^31^, ^32^ different screening tools might have disclosed different data. Additionally, future investigations with polysomnographic assessments are necessary to validate or contradict the findings of our study and others.^13^, ^14^, ^16^

Additionally, the myomucosal buccinator flap can be applied in a wide spectrum of patients with cleft palate and with velopharyngeal insufficiency, because aspects such as the types of previously performed palatine surgeries and/or the amount of scarring on the palate are not limiting factors for its indication. In fact, palatine elongation with the interposition of the bilateral buccinator myomucosal flap interrupts the restriction caused by the large amount of scarring on the palate (particularly the transition from hard and soft palates) by introducing healthy tissue with great elasticity in an area with poor vascularization secondary to previous scarring.^14^ Another theoretical advantage is that, as the maxilla is not connected to the posterior pharyngeal wall (as occurs in pharyngeal interventions), the buccinator myomucosal flap theoretically does not have a negative effect on facial growth when performed in patients with incomplete craniofacial development.

Among the disadvantages of the flap used in our study, we highlight the potential need for a second surgical procedure, although there is divergence about the need to perform this second one.^8^, ^9^, ^10^, ^11^, ^12^, ^13^ We have recommended pedicle division only in patients with mouth opening limitation and/or complaints related to chewing.

In the literature on velopharyngeal insufficiency,^1^, ^2^, ^26^ another relevant aspect is the postoperative time of follow-up. Most of the groups^14^, ^16^, ^17^, ^18^ performed the speech result measurements 3–6 months after the palatoplasty with the buccinator myomucosal flap. Although it has been shown that palatal edema decreases after 6 months postoperatively, palatine movement reaches an optimal level only 1 year after the surgery.^26^ Therefore, a period of at least 1 year should be considered the ideal for these evaluations. This is reinforced by our results, as there are significant differences in the measurements performed in the early and late postoperative periods.

Another group^13^ also showed speech results with more than 12 months postoperatively. However, the absence of speech results in intermediate periods (e.g., 3 months) does not allow comparative audiological and surgical therapeutic reasoning to be based on the understanding of the postoperative evolution of patients treated specifically with the buccinator myomucosal flap. Additionally, as Jackson et al.^33^ suggest that there is improvement in velopharyngeal function even after years of palatal surgeries, it is possible that subsequent evaluations (>12 months) of our patients could reveal distinct hypernasality results. Future studies should test this hypothesis.

Certainly, the technical details related to the handling of the palate and buccinator myomucosal flap vary between centers and surgeons.^13^, ^14^, ^15^, ^16^, ^17^, ^18^ Similarly to us, some use the bilateral flap to reconstruct the defect as anatomically as possible (to reconstruct the nasal and oral mucosae), while others use only the unilateral flap.^13^, ^14^, ^15^, ^16^, ^17^, ^18^ As another group,^18^ we believe that the lack of nasal mucosa reconstruction (i.e., surgical area in the nasal lining) can result in areas with inadequate vascularization, caused by wound healing by secondary intention. The scar contraction of the flap used in the oral mucosa reconstruction can culminate in the sagittal shortening of the palate. However, since we do not have objective data to support our hypothesis, it is important that future studies evaluate the influence of reconstruction with one or two buccinator myomucosal flaps on the hypernasality of patients with cleft palate.

Different authors^13^, ^14^, ^15^, ^16^, ^17^, ^18^ have discussed the safety and low rate of complications related to buccinator myomucosal flap use. Our complication rate (29.8%) is similar to the previously described trends (8–31.25%),^13^, ^14^, ^15^, ^16^, ^17^, ^18^ with differences in the studied populations and in data collection format. Interestingly, all of the complications reported here occurred in the first two years of flap use and in adult patients who had the third molars. Some groups^14^, ^16^ have performed modifications in the flap (e.g., placing the pedicle on the retromolar trigone or creating isolated flaps) or in postoperative care (for instance, bite blocks) to prevent patients from chewing pedicles and damaging blood flow. However, no comparative analysis was performed to test the efficacy of such modifications.^14^, ^16^ We believe that, as the main surgeon gains experience with the flap (i.e., careful dissection of the palatal tissue and flap, added to more detailed postoperative guidelines) these were enough to maintain the low rate of complications in the last two years analyzed. Although other groups^16^, ^33^ have also shown that the rate of complications decreased as the surgeon's experience increased, comparative future studies are required to verify whether any modifications (e.g., changes in pedicle position or use of bite blocks) will reduce complications.

We acknowledge that this study has some limitations. We have not used all existing tools or methods to assess speech outcomes, although all measurements were based on previously published methods or scores^8^, ^9^, ^10^, ^21^, ^22^, ^23^, ^24^, ^25^, ^26^, ^27^ and there is no consensus on the ideal methodology for of velopharyngeal insufficiency measurement.^1^, ^2^ It is important to emphasize that studies using objective assessment methods to analyze the degree of hypernasality (e.g., nasometry, which allows estimating speech resonance through nasalance measurement^4^, ^34^, ^35^), could show different results.

As in similar studies,^13^, ^14^, ^15^, ^16^, ^17^, ^18^ an additional limitation is the absence of a control group (e.g., pharyngeal flaps or sphincteroplasty). It is important to emphasize that our data are restricted to a selected subpopulation of patients with cleft palate (adolescents/adults, absence of palatine fistula, secondary or tertiary surgeries for the treatment of velopharyngeal insufficiency, moderate or severe hypernasality, and medium or large velopharyngeal gap). Although this subgroup reflects the reality of a reference center for cleft palate defect in Brazil,^39^ any generalizations should be made with caution. Moreover, as Mann et al.^16^ demonstrated satisfactory voice results exclusively in patients with a small velopharyngeal gap treated with the buccinator myomucosal flap, future studies should expand our indications, as well as confront and expand our results and verify whether our limitations interfere with the results.

## Conclusion

The buccinator myomucosal flap is effective in improving the velopharyngeal function of patients with cleft palate (± lip), particularly in reducing/eliminating speech hypernasality.

## Conflicts of interest

The authors declare no conflicts of interest.
